# The Riemannian Geometry Theory of Visually-Guided Movement Accounts for Afterimage Illusions and Size Constancy

**DOI:** 10.3390/vision6020037

**Published:** 2022-06-20

**Authors:** Peter D. Neilson, Megan D. Neilson, Robin T. Bye

**Affiliations:** 1 School of Electrical Engineering and Telecommunications, University of New South Wales, Sydney, NSW 2052, Australia; 2 Independent Researcher, Late School of Electrical Engineering and Telecommunications, University of New South Wales, Sydney, NSW 2052, Australia; megan.neilson@gmail.com; 3 Cyber-Physical Systems Laboratory, Department of ICT and Natural Sciences, NTNU—Norwegian University of Science and Technology, Postboks 1517, NO-6009 Ålesund, Norway; robin.t.bye@ntnu.no

**Keywords:** Riemannian geometry, visual space, visually-guided movement, afterimage illusions, association memory network, size constancy

## Abstract

This discussion paper supplements our two theoretical contributions previously published in this journal on the geometric nature of visual space. We first show here how our Riemannian formulation explains the recent experimental finding (published in this special issue on size constancy) that, contrary to conclusions from past work, vergence does not affect perceived size. We then turn to afterimage experiments connected to that work. Beginning with the Taylor illusion, we explore how our proposed Riemannian visual–somatosensory–hippocampal association memory network accounts in the following way for perceptions that occur when afterimages are viewed in conjunction with body movement. The Riemannian metric incorporated in the association memory network accurately emulates the warping of 3D visual space that is intrinsically introduced by the eye. The network thus accurately anticipates the change in size of retinal images of objects with a change in Euclidean distance between the egocentre and the object. An object will only be perceived to change in size when there is a difference between the actual size of its image on the retina and the anticipated size of that image provided by the network. This provides a central mechanism for size constancy. If the retinal image is the afterimage of a body part, typically a hand, and that hand moves relative to the egocentre, the afterimage remains constant but the proprioceptive signals change to give the new hand position. When the network gives the anticipated size of the hand at its new position this no longer matches the fixed afterimage, hence a size-change illusion occurs.

## 1. Introduction

In 2018 we published a theoretical paper in this journal on the Riemannian nature of visual space [1]. This was followed in 2021 by a further publication, again in this journal, in its special issue on size constancy [2]. This second theoretical paper built on the first to present a neurally feasible means for forming a visual–somatosensory–hippocampal associative memory underlying visually-guided movement. Subsequently, an experimental paper by Paul Linton appeared in the same special issue entitled *Does Vergence Affect Perceived Size?* [3]. In his review of the background to this work and in his discussion, the author mentions many earlier accounts of so-called ‘vergence micropsia’. There is particular reference to the Taylor illusion as well as to similar phenomena involving afterimage effects associated with movement. We believe that our Riemannian theory offers a cohesive explanation for these phenomena as well as accounting for Linton’s own finding that, contrary to considerable opinion otherwise, vergence does not affect the perceived size. The aim of this paper is therefore to detail this claim, finding by finding.

We realise that while Riemannian geometry has been used previously in other studies of visual space (see [1]) it is unknown territory for many biological and behavioural scientists. Indeed, aside from its application in physics, beginning notably with Einstein, it is not familiar territory for many mathematicians and engineers. So, before presenting our analysis of each set of findings, we offer a taste of the principles and of the way we have used these to develop our theory of visually-guided movement. These summaries are intentionally intuitive and have none of the mathematics given in our two previous papers [1,2] that can be consulted for deeper understanding. Indeed, for the reader wishing to see the mathematical underpinning of the verbal accounts given here, these papers [1,2] are essential reading.

In Section 2, we discuss Linton’s experiment in light of an introductory summary of Riemannian visual space. Section 3 concerns the Taylor illusion [4] and a number of related works involving afterimages and hand movement. Again, there is an introductory summary of the Riemannian association memory that we propose underlies visually-guided movement. We use this concept in analysing each of the results of the afterimage studies. In Section 4, we discuss the work of Zenkin and Petrov [5] whose findings on rich afterimages were not in keeping with Emmert’s law [6]. Again, these are analysed in terms of the Riemannian association memory and in particular its hippocampal component which enables place location. In the final section, we recap the theory and point to how it gives a unified explanation of the preceding body of experimental findings.

## 2. The Linton Experiment

We begin by summarising some aspects of the Riemannian theory pertinent to Linton’s conclusion that vergence does not affect perceived size.

### 2.1. Riemannian Background

From Listing’s law, it is well known that the size of the image of an object projected onto the retina varies in proportion to the solid angle subtended by the object at the nodal point of the eye. This implies that as the distance between an object in the environment and the nodal point of the eye changes, the size of the image on the retina changes. Likewise, the shape of the retinal image of an object changes as the object is viewed from a different angle. Thus, as a person moves about in a local 3D environment both the size and the shape of the retinal images of fixed objects change as a function of the place of the person’s egocentre in that environment, giving rise to optical flow. In 2018, we used the theorems of Riemannian geometry to deduce what these changes in size and shape of retinal images tell us about the geometry of 3D visual space [1]. The outcome was a warped 3D visual space described by the Riemannian metric g(r) where g(r) varies in inverse proportion to the square of the Euclidean distance r at each point (r,θ,φ) in the space, the origin being the egocentre, and (θ,φ) being the direction of gaze relative to an external reference frame. A key point here is that this description is mathematical, based on the anatomy and physiology of the eye, and is free of any modification by top-down cognitive processes that can confuse the description of visual space by subjective report.

To illustrate the Riemannian warping generated by the metric g(r), consider the example of looking at a brick wall in the fronto-parallel plane. As the gaze is shifted laterally along the wall, the bricks appear to decrease in size and assume the well-known trapezoidal shape associated with perspective. This can be attributed to two effects. Firstly, the angle subtended at the eye by a brick decreases as the distance of the brick along the wall increases. In other words, the angle between the brick and the direction of gaze decreases with distance as the brick appears more slanted to the line of sight. Secondly, the angle subtended at the eye by a brick decreases as the distance along the wall increases because the distance of the brick from the eye increases. So even if we corrected for the first effect by rotating each brick so that it became normal to the direction of gaze, the second effect would still make bricks that are further away appear smaller in size.

Both these effects along with the changing trapezoidal shape are intrinsic to the 3D visual space defined by the Riemannian metric g(r) at the points (r,θ,φ). We showed in 2021 [2] that if this metric is embedded in a partitioned memory spanning the points (r,θ,φ) the size and shape of an object at a particular (r,θ,φ) can be anticipated. If the predicted size and shape match what is observed, the object will not be perceived as changing in size or shape when seen from different places in the environment. This provides a mechanism for size (and shape) constancy. We will elaborate on this association memory in the subsequent section on afterimages, but first, we will use the Riemannian metric and the example of the brick wall to address Linton’s findings.

### 2.2. Linton’s Report

To foveate an object under normal viewing conditions the angles of the eyes in the head have to be adjusted so the visual axes of the eyes intersect precisely at the gaze point on the object. Linton [3] designed his experiment to test the long-believed notion that vergence (i.e., angular rotation of the eyes) plays a key role in size constancy. He constructed an apparatus that allowed the fixation distance to a target to be adjusted at the same time as the size of the target was altered. Specifically, participants viewed a fused target of angular size 3° which grew or shrank by between −20% and +20% over 5 s while at the same time the fixation distance specified by vergence was reduced from 50 cm to 25 cm. The changing fixation distance was achieved as follows.

Two identical target images a horizontal distance apart were arranged in a fronto-parallel plane such that the left eye was unable to see the left target and the right eye was unable to see the right target. Participants fused the two targets into a single image by converging the eyes. To achieve this, the visual axes of the two eyes have to cross at a point between the face and the fronto-parallel plane. The fused image thus appears to be located at this point (this is not dissimilar to depth perception in viewing random-dot stereograms [7,8]). The horizontal distance between the targets determines the necessary point of intersection of the visual axes and so by varying that distance the apparent depth of the fused image could be altered.

However, altering the lateral position of the targets in the fronto-parallel plane introduces a problem. When the left target is moved further to the left the change in angle subtended at the right eye decreases and likewise for the left eye when the right target is moved further to the right. Similarly, an angle increase occurs if the targets are moved towards the centre. To control for this Linton “rendered” the size of the targets to maintain a constant radius and a constant angle subtended at each eye despite changes in their horizontal position. To quote Linton directly: “… in order to present a constant retinal image with eye rotation, we rendered the targets to maintain a constant radius from, and orientation to, the eye. This was achieved in OpenGL by ‘projecting’ the target onto the display, so that the correct retinal image was achieved when the participants viewed the target …” [3] (p. 7).

There were other careful measures introduced in the experiment, but it is the above rendering that is of significance here. Using this apparatus Linton found no evidence that change in vergence affected participants’ judgements of target size. His methodology succeeded in dissociating variation of vergence from perception of size change and the result implied, therefore, that eye movements per se do not affect perceived size. We now suggest that in terms of the Riemannian theory this result is obtained simply because the rendering of the targets compensates for the warping of visual space introduced by the Riemannian metric.

### 2.3. Analysis

**Figure 1:**
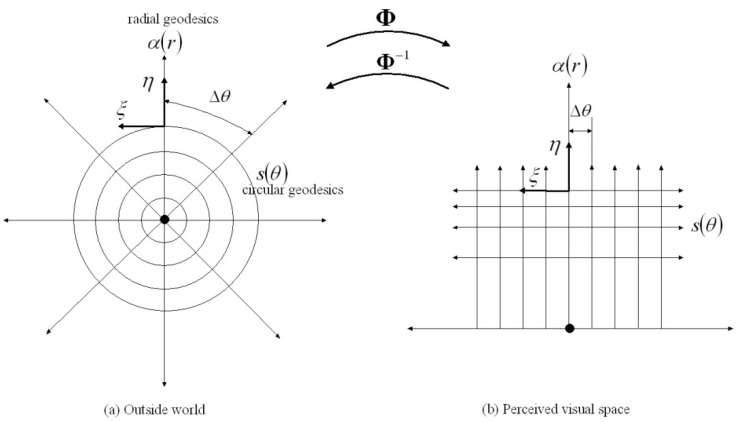
An illustration of the smooth conformal mapping Φ between (**a**) cylindrical coordinates (r,θ) on any plane in the 3D Euclidean outside world passing through the egocentre represented by the dot ● at the origin and (**b**) the corresponding plane in the warped Riemannian geometry of 3D visual space with the egocentre again represented by ●. Φ maps circular geodesics s(θ) and radial geodesics α(r) intersecting at right angles in the Euclidean outside world to corresponding horizontal straight lines s(θ) and vertical straight lines α(r) intersecting at right angles in the perceived visual space. The vectors ξ are Killing vectors whose integral flows preserve the metric g. The vectors $\eta =\dot{\alpha }$ are velocity vectors tangent to the radial geodesics α(r). $\Phi^{-1}$ depicts the inverse mapping from (**b**) to (**a**) and corresponds to the transformation applied by Linton. Notice that arc lengths s=rΔθ in the outside world increase linearly with Euclidean distance r while the corresponding spacing along the horizontal lines in visual space is constant. Also notice that a linear increase in radial distance r in the outside world corresponds to a logarithmic decrease in distance in visual space. The difference between the two coordinate systems illustrates the profound warping of visual space attributable to the anatomy and physiology of the eye. The warping is consistent with the perceived size of objects in visual space being proportional to the angle subtended at the egocentre by the object in the outside world.

In Section 4.3 of our 2018 paper on the warping of visual space [1], we used Riemannian geometry to show that (i) an object approaching with constant speed will appear to loom in size and accelerate as it nears the observer, and (ii) for an approaching object to appear to have a constant size and constant speed as it nears the observer it would have to shrink in size and decelerate by just the right amount to compensate for the visual warping. In other words, it is possible to manipulate events in the outside world so as to exactly counter the warping of 3D visual space introduced by the anatomy and physiology of the eye. This is exactly what occurred with the rendering of the targets in the Linton experiment. Put technically, in Section 5.6 of [1], we computed the conformal mapping Φ between the egocentric Euclidean geometry of the 3D outside world and the egocentric warped Riemannian geometry of 3D visual space. The inverse mapping $\Phi^{-1}$, from egocentric warped visual space back to the egocentric Euclidean outside world, is precisely the transformation that Linton applied to his targets. We illustrate these mappings in Figure 1.

To illustrate this more intuitively consider again the example of the brick wall in the fronto-parallel plane and the two effects discussed earlier that lead to size and shape distortion. Suppose now that this is not a real wall but a computer-generated image. Each brick in that image will present a constant retinal image when viewed laterally if it is rendered so as to maintain a constant radius from and orientation to the eye when the eye rotates. In other words, this changes the size and shape of the bricks so they appear to have constant size and shape as gaze is shifted horizontally along the wall. By enforcing this on his targets Linton ensured size constancy whatever the eye rotation, simply by cancelling the warping of visual space. We readily acknowledge that Linton’s careful experiment controlled expertly for a number of potential confounds. However, our point here is that the rendering employed in that context was the key to an essentially inevitable finding of size constancy being independent of vergence.

In the next section, we summarise our proposal of a Riemannian visual–somatosensory–hippocampal association memory and its role in providing size constancy [2]. The relevance here is that such a memory is built in the first place, and subsequently maintained, by means of adaptive modelling of the relationship between Euclidean depth r and the size of its associated retinal-hyperfield image during each fixed-gaze interval. The main mechanisms for estimating an absolute Euclidean depth r (prior to any top-down cognitive modifications of perceived depth) are stereopsis, retinal-image disparity and focus control. All three involve changes in vergence. Consequently, in this sense alone, it is not wrong to suggest that vergence plays a role in size constancy. This long-held belief is correct inasmuch as the influence of vergence can come indirectly via a central distributed memory mechanism as well as from possible direct effects of the type Linton was testing for. 

## 3. Afterimage Experiments

We begin by summarising the essentials of the distributed Riemannian visual–somatosensory–hippocampal association memory proposed in [2] to subserve visually-guided movement. This underpins our analysis of the curious effects reported in an assortment of afterimage experiments referenced by Linton [3]. Carried out between 1941 and 2017, these typically concern the way that afterimages, produced in the dark by a brief flash of light, change in response to subsequent unseen body movement.

### 3.1. Riemannian Background

While it is easy to postulate the existence of neural maps that associate visual, somatosensory and hippocampal inputs there are in fact many computational difficulties standing in the way of the nervous system actually being able to form such maps. The complications include the following: (i) nonlinearities warp both visual space and proprioceptive space, (ii) any visual–proprioceptive mapping will be highly redundant with at least 73 joint angles influencing the perceived position of the body in 3D visual space, (iii) gaze points on the surface of the body are occluded from view in some postures but not in other postures, (iv) the visual axis of the eye does not pass through the centre of rotation of the eye, (v) the position, size and shape of 2D images on the retinas are determined by the solid angle subtended by the 3D object in the environment at the nodal points of the eyes creating disparity between left-eye and right-eye retinal images, (vi) changing the place of the head in the environment or changing the posture of the body each cause a change within 3D visual space of the position, size, shape, curvature, outline, occlusions, relative velocity and relative acceleration of encoded retinal-hyperfield images of objects and/or of the body in that environment, (vii) mass-inertia loads about each elemental movement of the body change with both changes in posture of the body and with changes in mechanical interactions between the body and the environment. 

The Riemannian geometry theory of 3D binocular visual perception and visually-guided movement described in detail in [1,2] takes these complications into account and presents a neurally feasible means of forming such a distributed association memory network. Moreover, it shows that this network can generate a smooth, one-to-one, onto, invertible map between the posture of the body sensed proprioceptively and the visual image of the body in warped 3D visual space. The distributed association memory network partitions visuospatial memory into sub-memories each associated with (i) a given posture of the body encoded proprioceptively in the somatosensory cortex and (ii) the place of the head (egocentre) in the 3D environment encoded by place cells in the hippocampal formation. We see the hippocampal formation, subiculum and entorhinal cortices as forming an innate representation of 3D Euclidean space anchored to the outside world by vestibular afferent signals and visual landmarks. Each partition of visuospatial memory provides a representation of a warped gaze-based 3D visual space. The warping of 3D visual space is the same in each partition and is encoded by the Riemannian metric g(r) consistent with the size of retinal images varying in inverse proportion to the Euclidean distance between the nodal point of the eye and the object in the environment.

During each fixed-gaze interval, encoded retinal-hyperfield images are stored into the appropriate partition of visuospatial memory associated with the current posture of the body sensed proprioceptively and with the current place of the head in the environment encoded within the hippocampus. Each gaze-based sub-memory is itself an association memory network in which encoded retinal-hyperfield images from each fixed-gaze interval are associated with their cyclopean coordinate vector (r,θ,φ) where r equals the Euclidean distance between the egocentre midway between the eyes and the point in the environment that projects to the given retinal hyperfield and the angles (θ,φ) give the direction of the cyclopean vector measured with respect to an external reference frame (X,Y,Z) provided by the hippocampal formation.

To illustrate the value of such a distributed Riemannian visual–somatosensory–hippocampal association memory network, consider the case of a tennis player about to return a tennis ball. The player can keep their ‘eye on the ball’ and does not have to look at their own body to determine where the different parts of the body are located in 3D space. Given the current proprioceptive and vestibular afferent signals, the distributed association memory network informs the response planning parts of the brain about the current place of the egocentre on the tennis court and the current visual image of the body in 3D visual space. This is essential information needed to plan, in a single reaction-time interval, a multi-joint coordinated movement from the current posture and velocity of the body to the required configuration of the body for the racquet to strike the ball in a specified fashion.

We now consider how the existence of such a memory might explain the results of the various afterimage experiments that follow.

### 3.2. The Taylor Illusion

In 1941, the following now well-known phenomenon was reported by Taylor [4]. If, in the dark, a bright flash of light forms a positive afterimage of a person’s hand held at arm’s length in front of the face and then, in the dark, the person moves that hand towards the face, the afterimage of the hand appears to decrease in size. Alternatively, if the hand is moved away from the face in the dark, the afterimage appears to increase in size. Likewise, if the person holds the position of the hand fixed but moves the head towards (or away) in the dark, the afterimage similarly appears to decrease (or increase) in size. 

#### Analysis

As a person moves a hand towards the face, the Euclidean distance r between the egocentre and the gaze point on the hand decreases. The Riemannian metric g(r) at the (r,θ,φ) memory site for the new hand position (sensed visually and proprioceptively) increases because the metric (i.e., the distance between points) varies in inverse proportion to the Euclidean distance r. As a consequence, the visual image of the hand retrieved from the distributed Riemannian visual–somatosensory–hippocampal association memory network anticipates the increasing size of the encoded retinal-hyperfield images. Under normal viewing conditions, the anticipated image and the actual image of the hand match. Thus, the Riemannian association memory network provides a mechanism for size constancy.

Exactly the same mechanism is in play in the Taylor illusion when a person looks at the afterimage of the hand while the actual hand is moved towards the face in the dark. The new hand position (r,θ,φ) can only be sensed proprioceptively, but this is sufficient for the association network to give the anticipated larger retinal image. However, in this case, the “actual” image is the afterimage that is fixed in size on the retinas. So, when the hand approaches the face, the anticipated increase in the size of the available visual image does not occur and the hand, as perceived in the afterimage, appears to decrease in size. The reverse applies when the hand is moved away from the face. The anticipated decrease in the size of the available visual image does not occur and the afterimage of the hand appears to increase in size.

The same basic reasoning obtains when the head moves towards or away from a fixed hand. The end situation is the same as above with the Riemannian metric g(r) at the (r,θ,φ) memory site for the new head position altering because the metric varies in inverse proportion to the Euclidean distance r. However, here, the situation is more complicated than with a hand movement where the only proprioceptive change may be that involving the elbow joint. A changed head position can only be produced by a change in posture of the body involving multiple elemental movements. Nevertheless, the Riemannian association memory network is such that it accommodates whole body proprioception [2] and so the appropriate anticipated visual images will be retrieved for comparison with the actual images on the retina. Thus, the Taylor illusion is explained, whether it is the hand that moves, or the head, or indeed both.

In summary, the Riemannian theory predicts that an apparent change in the size of the hand (or its afterimage) will be perceived whenever a difference occurs between the size of the retinal image of the hand and the size of that image anticipated in visual–somatosensory–hippocampal association memory from the current position (r,θ,φ) of the hand in the egocentric 3D visual space.

### 3.3. The “Crumble” Effect

In 1973, Davies [9] studied the afterimage of two hands held side by side when one of the hands was removed in the dark. He found that only the afterimage of the removed hand changed, most often disappearing, while the afterimage of the other hand remained unchanged. He coined the term “crumble” effect to describe the part of the afterimage, usually that of the hand and arm that was removed, just disappearing in an otherwise unchanging positive afterimage. 

#### Analysis

According to the Riemannian geometry theory, the change in the encoded visual image in 3D space of any part of the body, including the hand, that occurs with a change in body posture can be profound. Its position, size, shape, outline, curvature, occlusions, relative velocity and relative acceleration can all change. However, when the afterimage of an outstretched hand is fixed on the retinas, only a subset of all the possible changes in posture in the dark are compatible with a rescaling of the size of that afterimage. For example, pronation of the hand under normal conditions will occlude all the gaze points on the palm of the hand and replace them with gaze points on the back of the hand. If a pronation movement of the hand is made in the dark while the person looks at an afterimage of the palm of the hand, then there is no way the afterimage can be rescaled to match the anticipated pronated image of the hand retrieved from the distributed Riemannian association memory network. The afterimage of the palm of the hand simply disappears; the “crumble” effect.

### 3.4. Disappearance of Afterimages at Impossible Locations in Space

In 1984, Hayhoe and Williams [10] reported that an eccentrically positioned afterimage viewed in the dark disappears if the eye is rotated to a position that projects the afterimage to a location outside the field of view framed by the brow, cheek and nose.

#### Analysis

By means of our Riemannian association memory network, we propose that the nervous system stores place-encoded retinal-hyperfield images for each cyclopean gaze point (r,θ,φ) on fixed objects as seen with the head in a fixed place. Orientation of the head is absorbed into the cyclopean gaze coordinates (r,θ,φ). Gaze points that cannot be seen from any place, such as points on the surfaces of objects pushed together, are not included. By the same argument, gaze points outside the visual field framed by the brow, cheek and nose with the head at a particular fixed place and orientation are never experienced and so are not included in the association memory network for that place and orientation of the head. This is the case in the Hayhoe and Williams experiment. The retinal-hyperfield images of gaze points (r,θ,φ) outside the framed field of view have not been experienced. Consequently, when the eyes are rotated in the dark to gaze at points outside that framed field while looking at an afterimage object, the anticipated afterimage disappears.

### 3.5. Proprioceptive Feedback Is Sufficient to Create the Taylor Illusion

In 1996, Carey and Allan [11] observed that passive and active movements of the arm and hand in the dark produced equivalent illusory size changes in the afterimage. They concluded that proprioceptive feedback is sufficient to drive the illusion without the need for efference copy (i.e., copy of outgoing motor commands). They also found that the afterimage of the non-dominant hand placed in front of the dominant hand so as to occlude it from view during the flash of light did not change in size when the dominant hand was moved away from the non-dominant hand in the dark.

#### Analysis

As detailed in the Riemannian theory [1,2] and in previous descriptions of adaptive sensory-motor control [12,13], to initiate an appropriate pattern of outgoing motor commands to achieve a specified visual goal the inverse nonlinear dynamical transformation from the specified visual goal to the required outgoing motor commands has to be performed. Because of time delays, nonlinearity and redundancy in the motor system (i.e., many more descending motor commands than elemental movements) no unique inverse relationship exists. It is analogous to trying to solve a system of nonlinear dynamical simultaneous equations with more unknowns than equations. The problem of time delay, nonlinearity and redundancy can be overcome using prediction and, as described in [2], by selecting a particular nonlinear multi-joint coordination to achieve the visual goal with redundancy removed. Indeed, one could say that the main point of the theory presented in [2] is to show how such a goal-dependent minimum-effort movement synergy exists. Having removed redundancy by selecting an appropriate goal-dependent movement synergy (i.e., multi-joint coordination) [13] it then becomes possible to compute the required goal-dependent inverse transformation between the specified visual goal and the required coordinated pattern of outgoing motor commands, a computation that has to employ efference copy [2,12].

While the above description stresses the importance of efference copy in active control of goal-directed movement, the actual posture of the body, regardless of whether it is actively or passively determined, is nevertheless encoded by proprioceptive afferent signals independently of efference copy. As emphasised in the Riemannian theory, it is the distributed Riemannian visual–somatosensory–hippocampal association memory network with posture encoded proprioceptively that underlies size constancy. Thus, consistent with the finding of Carey and Allen [11], the Riemannian theory predicts that the afterimage illusion will occur regardless of whether the arm and hand are moved either passively or actively in the dark.

The theory also predicts the further finding concerning the occlusion of one hand by the other. When the dominant hand is occluded by the non-dominant hand during the bright flash of light only the non-dominant hand is represented in the resulting retinal afterimage. The movement of the dominant hand away from the non-dominant hand in the dark sensed proprioceptively is not associated with movement of the dominant hand in the Riemannian association memory. Consequently, movement of the dominant hand in the dark has no effect on the apparent size of the afterimage of the non-dominant hand.

### 3.6. Vergence Movements of the Eyes Explain the Taylor Illusion

In 1997, Mon-Williams and colleagues [14] argued that arm movements alone are not sufficient to cause the perceived size change of retinal afterimages in the dark. They showed experimentally that changing limb position in the dark alters vergence angles of the eyes and they explained the illusion using this mechanism. Subsequently, in 2013, Sperandio and colleagues [15] used behavioural and eye-tracking experiments to demonstrate that while perceived changes of afterimages in the dark were determined mainly as a function of the vergence, the strength of this relationship decreased when a mismatch between vergence and proprioceptive hand position was introduced. They concluded that under restricted viewing conditions the visual system relies on multimodal signals to gather information about distance and make inferences about object size.

#### Analysis

As detailed in [2] and mentioned in Analysis section in Section 3.5, a purposive movement that involves looking at the hand as it is moved about in 3D space requires the selection of a task-dependent minimum-effort movement synergy (or sequence of movement synergies). Such a task-dependent synergy is a coordinated multi-joint movement selected to achieve the given goal. The synergy must be selected before goal-directed trajectories within that movement synergy can be planned. If the goal is to visually track the hand as it is moved about in 3D space then the selected task-dependent movement synergy will include coordinated movements of the hand, head and eyes.

If a person imagines performing this task in the dark while looking at an afterimage of the hand, then movement of the hand will also include coordinated vergence movements of the eyes. However, there is no reason a person cannot imagine performing a task involving movement of the hand while directing their gaze at some other point away from the hand. For example, a tennis player can keep their eye on the ball while planning and executing a multi-joint coordinated movement of the body to return the ball. In other words, according to the Riemannian theory, an afterimage of the hand can change size in association with movement of the hand in the dark both with or without an associated vergence of the eyes depending on the task the person imagines performing.

### 3.7. A Limit on the Decrease in Size of the Afterimage

In 2000, Bross [16] remarked that the most intriguing observation on the afterimage of the hand was that there appears to be a limit on the decrease in its perceived size. The visual system ‘refuses’ to size-scale the hand below a limit it accepts as representable or acceptable of ‘its’ hand.

#### Analysis

If a person looks at their hand under normal conditions, then as it is moved closer to the face the size of the image projected onto the retinas increases in inverse proportion to the Euclidean distance r between the hand and the egocentre. Such an inverse function accelerates towards infinity as r approaches zero. When the hand reaches a few centimetres from the face its image on the retinas exceeds the field of view framed by the brow, cheek and nose. Only a portion of the hand projects an image onto the retinas and different patches of the hand are projected onto the left-eye and right-eye retinas. When the hand is moved in the dark, there is no way a rescaling of the fixed afterimage of the hand on the retinas can compensate for such a complicated proprioceptive-to-visual relationship stored in the distributed visual–somatosensory–hippocampal association memory network. This imposes a limit on the size of rescaling.

### 3.8. Displacing the Visual Image 10° Left Has No Effect on the Taylor Illusion

In 2007, Ramsay, Carey and Jackson [17] hypothesised that the visual and proprioceptive information has to be in strict “register” when the afterimage is formed in order for the Taylor illusion to occur. They suggested that this can explain why the afterimage illusion did not occur for the non-dominant hand in the Carey and Allan experiment [11] described in Section 3.5.

To test this hypothesis, participants performed ‘towards’ and ‘away’ movements of a card held in the dominant hand after obtaining afterimages of the card when the participants wore glasses with either plain lenses or prism lenses displacing the visual image 10° to the left. They reasoned that if the subtle “mismatch” between visual and proprioceptive information regarding the target’s objective position is indeed responsible for the failure of the Taylor illusion in the earlier experiment, then the gross mismatch of the position signals when prism glasses are worn should null the illusion even more completely. The result of the experiment showed no significant effect of the lenses. Despite the visual image being shifted by 10° to the left when some participants formed the afterimage of the hand-held card, the Taylor illusion for this afterimage still occurred for both ‘forward’ and ‘away’ movements of the card in the dark.

#### Analysis

Using prism glasses to shift the image of the card held in the dominant hand by 10° to the left when the afterimage of the card is formed creates a mismatch between the gaze vector (r,θ,φ) and the proprioceptive coding of the position of the dominant hand holding the card. It is this lack of register between the position of the card sensed visually and the position of the hand sensed proprioceptively that the authors hypothesized should null the Taylor illusion when the dominant hand holding the card moves in the dark. However, this mismatch is confined to (θ,φ) encoding the direction of gaze with the image shifted by 10° to the left while the Euclidean distance r remains more or less unchanged. The Riemannian metric g(r) at the associated (r,θ,φ) memory site proposed in the Riemannian theory to warp 3D visual space is a function of r alone and independent of the direction of gaze (θ,φ). Consequently, warping of 3D visual space is spherically symmetrical about the egocentre. The decrease in size of retinal images with increasing r is the same along all radial lines extending outwards from the egocentre. Thus, a shift in the direction of gaze by 10° will have no influence on the anticipated change in size of the retinal image of the card held in the dominant hand.

### 3.9. Perception of Afterimages Depends Not Only on Bodily Signals but Also on the Sense of Self

In 2017, Faivre and colleagues [18] conducted an experiment in which an afterimage projected onto a participant’s right hand drifted laterally toward a rubber hand only when the rubber hand was embodied. Participants sat in the dark with crossed left and right hands resting on a desk. An array of LEDs was flashed to produce an afterimage of the participant’s right hand. When this became stable, an experimenter induced the rubber hand illusion by stroking a right rubber hand with the participant’s left index finger while simultaneously synchronously stroking the corresponding part of the participant’s right hand. After about 10 s of stroking the participant obtained the illusion that their left hand was stroking their right hand. They then reported that the afterimage of the right hand appeared to drift laterally towards the rubber hand.

#### Analysis

In Section 8.8 of [2], we pointed to how the Riemannian theory can be extended to include integration not only of posture and place with 3D visual space but also with other sensory modalities, in particular with tactile and auditory spaces. A Riemannian association memory network extended to include tactile inputs can explain the results of the rubber hand experiment.

For the participant to obtain the tactile illusion that their left hand is stroking their right hand when in fact the two hands are some distance apart on the desk a recalibration of the proprioceptive signals has to occur. Either the proprioceptive sense of the position of the left hand has to move laterally to the left or the proprioceptive sense of the position of the right hand has to move laterally to the right, or a combination of the two. It would seem from the reported illusion that adaptive retuning of sensory-sensory maps within the nervous system can happen very quickly, in as little as ten seconds. If we extend the Riemannian theory to include multimodal sensory information and allow for very fast sensory-sensory adaptation, retuning of proprioceptive to tactile maps can account for the apparent lateral shift of the right-hand afterimage towards the rubber hand.

## 4. The Zenkin and Petrov Experiment

### 4.1. Zenkin and Petrov’s Report

In 2015, Zenkin and Petrov [5] reported an afterimage experiment that relates both to the Taylor illusion and to Emmert’s law. It is not the typical afterimage experiment and so we consider it separately. These authors studied the influence of ten different eye and body movements on the afterimages of (i) a small rectangular white card and (ii) an entire 3D experimental room. They referred to these as a poor afterimage and a rich afterimage, respectively. These were viewed in conjunction with luminous points that were aligned in a frontal plane or in depth with a distance of 30 cm (about 12°) between neighbouring ones. Once the afterimage was formed participants were instructed to fix their gaze on one of the luminous points and then to perform one or more of ten different eye and body movements. We consider here findings from only the first four of these movements: (1) changing the fixation point in the frontal plane, (2) changing the fixation point in depth, (3) tracking a fixation point moving in the frontal plane, and (4) tracking a fixation point moving in depth. All involved changes in fixation and these were the only movements of the ten for which differences were observed between poor and rich afterimages. The exact findings are best summarised in the author’s own words:

*Change in the apparent position of the afterimages in space, caused by shifting the gaze to another fixation point in the frontal plane, occurred only for the poor afterimage, whereas the rich afterimage appeared constant in position … In these two situations, the objectively stationary luminous spots appeared to behave in the opposite way: They remained stationary in the case of the poor afterimage … and jumped in the opposite direction in the case of the rich one … Likewise, in the case of shifting the gaze to another fixation point in depth … the same difference between rich and poor afterimages was observed … In particular, as concerns the afterimages, an abrupt shift in depth to the new fixation point and change in size, depending on change in convergence, was noticed in the case of poor afterimages whereas in the case of rich afterimages, there were no changes either in position or size. Within the rich afterimage, the luminous points appeared to change in position and size.*

*Similar transformations were also observed in the case of pursuit eye movements. The poor afterimage moved in space with the tracking fixation point, while the rich afterimage remained in place under all circumstances. In the latter case, the tracking fixation point was also perceived as stationary (although it was moving at a speed of 10° per second to angles up to 40°), while the objectively fixed luminous spots were perceived as moving in the opposite direction … Particular attention was drawn to the fact that, in the case of a rich afterimage, a change of the fixation point in depth leads to an apparent change in size of real constant luminous spots …*

*This phenomenon constitutes a very impressive illusion and might be called **the illusion of a rich afterimage**. (authors’ emphasis)* [5] (p. 978)

While the findings for the poor afterimage conform, those for the rich afterimage are inconsistent with Emmert’s law. We argue in the next section that the difference lies in the fact that the rich afterimage contains sufficient information to generate a sense of the place of the egocentre within that afterimage.

### 4.2. Analysis

Consider first what happens when a person fixes their gaze on one of the luminous points under normal viewing conditions. An image of the small white card is projected onto peripheral regions of the left-eye and right-eye retinas. Despite the disparity of these retinal images visual, proprioceptive and vestibular afferent signals provide sufficient information for the nervous system to determine the cyclopean coordinates (r,θ,φ) for the position of the white card in 3D space with respect to an external reference frame (X,Y,Z) encoded within the hippocampal formation (for detail see [2], Section 2.7 and 2.8). The Riemannian metric g(r) warps the 3D visual space in anticipation of retinal images changing size in inverse proportion to the Euclidean distance r. Now suppose the person shifts their gaze to one of the other luminous points in the room without changing the place of the head. This involves a rotation of the eyes in the head, or a rotation of the head in the room, or a combination of both. Such a change causes the positions of the images of the small white card projected onto the peripheral retinas of the left eye and right eye to change. Again visual, proprioceptive and vestibular afferent signals provide sufficient information for the cyclopean coordinates (r,θ,φ) for the fixed position of the white card in 3D space relative to the external reference frame (X,Y,Z) to be determined. This accounts for the easily verified fact that shifting gaze from one point to another in 3D visual space does not change the perceived positions of objects in that space.

Now consider what happens when the luminous points are seen as part of an afterimage. In the case of the so-called poor afterimage, this was created by a bright flash of light on the small white card while the participant was fixating one of the luminous points. Accordingly, images of the card are “burnt” onto disparate parts of the peripheral retinas of the left and right eyes. As described above, the nervous system is able to determine the cyclopean coordinates (r,θ,φ) relative to the external reference frame (X,Y,Z) giving the 3D position of the white card in the room. However, when the person shifts their gaze in the dark to another luminous point in the room, the positions of the images “burnt” onto the retinas do not change. While the visual information is unchanged, proprioceptive and vestibular afferent signals have changed so the associated cyclopean coordinates $\left(r^{\prime },\theta^{\prime },\varphi^{\prime }\right)$ relative to the external reference frame (X,Y,Z) giving the 3D position of the white card in the room are now different. The poor afterimage of the card, therefore, appears to shift abruptly from one position in 3D visual space to another. If the change in fixation involves a change in depth, then warping of 3D visual space encoded by the Riemannian metric $g\left(r^{\prime }\right)$ causes a change in the apparent size of the poor afterimage. It reduces under convergence of the eyes and increases under divergence of the eyes just as for the Taylor illusion. Retinal images of real luminous points in the room, on the other hand, do change their positions on the retinas in association with a shift in fixation from one real luminous point to another. Consequently, the cyclopean coordinates (r,θ,φ) relative to the external reference frame (X,Y,Z) giving the 3D positions of the luminous points in the room do not change. Just as described above, shifting gaze from one real luminous point in the room to another does not change the apparent positions of any of the real luminous points in the room.

Now consider the effect of changing the fixation point from one luminous point to another in the rich afterimage. Remember, Zenkin and Petrov found that the rich afterimage behaved in a completely opposite way from the poor afterimage in response to changes in fixation. Their “rich afterimage illusion” is different in character from the Taylor illusion and is inconsistent with Emmert’s law. The question is, can the Riemannian geometry theory account for the rich afterimage illusion?

Since the rich afterimage was a voluminous 3D image of the entire room, the visual landmarks used by the hippocampal formation to anchor its innate representation of the 3D Euclidean space to the outside world are contained within the rich afterimage. In other words, the rich afterimage contains sufficient information to generate a sense of the place of one’s egocentre within the 3D afterimage. Cyclopean coordinates (r,θ,φ) to each point in the 3D-rich afterimage are determined with respect to this sensed place of the egocentre. The real luminous points are located within the 3D-rich afterimage. Shifting gaze from one point to another within the 3D afterimage leaves the cyclopean coordinates (r,θ,φ) to each point in the 3D-rich afterimage measured with respect to the sensed place of the egocentre in the rich afterimage unchanged. However, projections of the real luminous points onto the left and right retinas do change when the gaze is shifted from one real luminous point to another. Consequently, the cyclopean coordinates (r,θ,φ) of all the real luminous points measured with respect to the external reference frame (X,Y,Z) derived from landmarks in the 3D-rich afterimage all change in the direction opposite to the new fixation point. If the change in gaze involves a change in depth then, with respect to the external reference frame (X,Y,Z) derived from landmarks in the 3D-rich afterimage, all the real luminous points appear to increase in size as they move closer.

The same explanation holds when tracking a real moving fixation point, except that now the tracking fixation point appears to remain stationary because its image is held fixed on the foveas. The projections of other real luminous points drift away from the foveas so appear to be gradually shifting in the opposite direction. When this drift involves a change in depth, the apparent size increases as it approaches and decreases as it recedes. 

The other six eye and body movements studied in the experiment did not involve a change in fixation and produced no differences between effects on poor and rich afterimages, as is predicted by the Riemannian theory. Hence the theory is consistent with the findings for all ten eye and body movement conditions for both types of afterimage. Indeed, certain aspects of the Riemannian theory, such as the volumetric nature of 3D visual space with objects appearing to decrease in size with increasing Euclidean depth, are strongly confirmed by the experiment. 

Zenkin and Petrov concluded that visible transformations arise as a result of activity in the mental domain. In their view, we have to assume that a visible afterimage is not a simple copy of a retinal picture but is the product of activity of some intermediate level of the visual system. This is not all that different from our proposals. According to the Riemannian theory, it is not vergence or proprioception or vestibular afferent signals alone that are responsible for afterimage effects but rather it is the nonlinear dynamical relationships between those signals stored within a central distributed visual–somatosensory–hippocampal association memory network that provides the mechanism for size constancy.

## 5. Closing Commentary

Artists, philosophers and scientists have been interested for centuries in the relationship between the geometry of perceived 3D visual space and the Euclidean geometry of the 3D world. A difficulty is that there is no way the perception of other people can be measured experimentally other than having them report their perceptions in one way or another. Such reporting almost certainly involves cognitive processes that might modify the geometry of the visual space encoded by sensory signals. This can account for the many inconsistencies in reported experimental attempts to measure the geometry of visual space. The Riemannian geometry theory [1,2] was developed in order to overcome this dilemma by mathematically deriving the geometry of 3D visual space based on sensory signals before it is modified by top-down cognitive processes.

The resulting theory is concerned therefore with the geometry of 3D visual space encoded by visual, proprioceptive and vestibular afferent signals before cognitive mechanisms of depth perception come into play. The human visual system has evolved to take advantage of a frontal-looking binocular anatomy with high acuity central (foveal) vision. However, in doing so, it has had to cope with the inevitable size–distance relationship and warping of retinal images associated with such a binocular anatomy and physiology. To survive in a changing and uncertain 3D environment, it would seem important that the perceived 3D visual space should match as closely as possible the Euclidean structure of the outside world. To achieve this processing, the nervous system has to be able to anticipate and compensate for changes in the size and shape of 2D retinal images associated with changes in depth and orientation of objects in the environment. The Riemannian visual–somatosensory–hippocampal association memory network proposed in the Riemannian geometry theory achieves exactly this.

The resulting transformation of 2D retinal images into a 3D visual space is faithful therefore to the images that are actually projected onto the retinas. Actual retinal images are important because they provide the only link between visual events in the outside world with neural encoding of those visual events within the nervous system. If the sources of these retinal images are modified, say by rendering or if they are the result of afterimage formation, the encoding and the transformations from that encoding remain true to those actual retinal images. The associations of actual retinal images within our proposed adaptive visual–somatosensory–hippocampal association memory network will thus provide a means to predict the sensory consequence of any experimental manipulation of those images. Effectively, these associations specify a pre-conscious 3D Riemannian space linking vision to other correlated sensory input.

We now touch briefly on the relationship of our theory to certain others. The notion of a pre-conscious 3D Riemannian sensory visual space defined in an association memory network (Section 3.1 and [2]) is similar to the Bayesian [19] and empirical [20,21] statistical accounts of conscious visual perception inasmuch as the formation of adaptive neural filters based on least mean square algorithms can be seen as being similar to the learning of probability distributions and perceptual statistics. Beyond that, however, the theories differ considerably. Essentially the Riemannian formulation operates pre-cognitively based on direct encoding of sensory signals and adaptive-filter maps between signals while the empirical formulations operate cognitively relying on the statistical accumulation of visual information and probability distributions to transform retinal images into stable and predictable representations of the external world.

On the one hand, recapping from [1,2], the Riemannian theory holds that there is only one veridical geometry of 3D visual space, namely that encoded directly by visual, proprioceptive and vestibular afferents informing on the current visual scene in association with the current posture of the body and the place of the head in the local environment. A purely sensory encoding of the distance to visual objects is obtained via stereopsis, retinal-image disparity and focus control mechanisms before it is influenced by top-down cognitive mechanisms. Through visual scanning, an image of the 3D environment and of the body in that environment as seen from each fixed place and with the body in each fixed posture is formed in each partition of visuospatial memory. Formation of adaptive maps between each and every partition of visuospatial memory constructs a 3D representation of visual space and of the body in that space as seen from any place in the environment and with the body in any posture. It is this 3D visual space that we have shown mathematically to be a warped 3D manifold endowed with a Riemannian metric that accounts for the fact that the size of images projected onto retinal hyperfields change in inverse proportion to the Euclidean distance r between the nodal point of the eye and the point (r,θ,φ) in the environment projecting onto that hyperfield. Thus, the warping of the 3D Riemannian visual space anticipates changes in the size and shape of retinal images associated with changes in the place and posture of the body. We have also shown mathematically that despite a similar warping of proprioceptive-posture space caused by nonlinear mass–inertial interactions between the elemental movements of the body and mechanical interactions between the body and the environment, there exists a smooth, one-to-one, onto, invertible map between the posture of the body sensed proprioceptively and the visual image of the body in the 3D Riemannian visual space [2]. For this reason, we suggest that it is this pre-conscious 3D Riemannian visual space that subserves the planning and execution of visually-guided movement.

In contrast, the empirical view of visual perception depends on “knowledge” of statistical probability distributions of natural visual scenes accumulated over time through experience. Since the visual system has no direct measure of depth, it is confronted by the problem recognised by Helmholtz that retinal images are ambiguous because variations in viewpoint and lighting can cause different objects to have similar retinal images and the same object can give rise to different retinal images [19]. Moreover, where binocular stereopsis and retinal-image disparity mechanisms of depth perception are not available (such as in monocular vision, in seeing depth in pictures or for objects too far away), the visual system must resort to top-down cognitive mechanisms of depth perception. A large variety of these mechanisms exist and can involve occlusions, relative size, texture gradients, shading, height in the visual field, aerial perspective and perspective. These mechanisms all depend on statistical “knowledge” acquired from previous experience. According to Howe and Purvis [20] (p. 13188), “the strategy of vision that best can ensure appropriate visually guided behavior in response to retinal stimuli of uncertain provenance would be to generate percepts according to the probability distributions of the possible sources.” However, how such percepts link to the appropriate behaviour seems unclear. The Bayesian statistical decision theory based on the conditional probability of a hypothesis (percept) given the outcome event (retinal image) is described by Geisler and Kersten [19] as providing a framework for optimally interpreting ambiguous retinal images. Just as in the Howe and Purvis account the prior probability and likelihood distributions incorporated in the visual system arise through a combination of evolution and perceptual learning of natural scene statistics. 

To us, a problem with visual percepts derived from the learned statistics of natural scenes is that the probability distributions are almost always strongly non-Gaussian. This indicates the existence of at least second-order and third-order nonlinearities (viz., skewness and kurtosis) in detecting features such as lengths of slanted straight lines and angles between lines. Straight lines on the retina may well be generated by curved lines in 3D space. The assumption of Gaussianity in many statistical procedures, including Bayesian ones, can be vexed. More importantly, probability-based estimates almost always lead to percepts that deviate from those obtained directly from visual, proprioceptive and vestibular sensory signals. These deviations change the geometry of the perceived 3D visual space and can lead to illusions in visual perception. While such learned top-down cognitive mechanisms of depth perception can transform rapidly retinal images into enhanced perceived perceptions of the 3D world, at the same time those consciously perceived images are almost certainly populated with all sorts of deviations from reality. Magicians take advantage of such visual illusions to trick the perceptions of their audience. It should not be surprising then that statistical empirical theories, operating as they do at cognitive levels, account nicely for many visual illusions. On the other hand, illusion-laden perceptions do not provide a good basis for the planning and control of visually-guided movement. Statistical theories do not do a good job at accounting for the infinite number of possible motor actions “afforded” by a visual perception. They do not readily explain the experimentally observed afterimage phenomena presented in this paper. Nor do they address the perception-action dissociations reported elsewhere [22,23,24,25] and examined by us previously in light of the Riemannian theory [2] (Section 8.7).

We hold that whenever top-down cognitive mechanisms of depth perception overrule estimates of depth obtained from stereopsis, retinal image disparity and/or focus control (i.e., the estimates obtained directly from sensory signals) the geometry of 3D visual space is changed. Depth-dependent illusions are introduced into visual perception. The smooth, one-to-one, onto, invertible map between the posture of the body sensed proprioceptively and the encoded visual image of the body in warped 3D visual space is lost. We argue therefore that it is the intrinsic Riemannian geometry of 3D visual space before it is modified by top-down cognitive mechanisms of depth perception that is employed by the nervous system for the planning and execution of visually-guided movement. This accounts for the dissociation between illusions attributable to top-down cognitive processes and actions observed experimentally as well as for the existence of “blind sight’ described by Goodale and Milner [26,27].

These observations suggest that experiments aimed at measuring the geometry of 3D visual space before it is perturbed by top-down cognitive mechanisms should focus on the measurement of visually-guided movement rather than on the subjective reporting of perceptions. We suggested in [2] that the dissociation observed experimentally between perception and action in illusions may provide an excellent method for measuring differences between the geometry of a pre-conscious sensory visual space and the geometry of the final conscious perception. We need more experiments where a movement links to the nature of the warped pre-conscious visual space rather than to the conscious visual perception.

When applied as in this paper, the Riemannian theory offers a unified explanation for an apparently diverse range of experimental observations on afterimages and illusions. This adds consistently to the way in which it is pertinent to a variety of phenomena concerning how we see the world. As set out previously in [1], these include: (i) ability to form a 3D representation of the visual environment seen in the correct perspective from any place in the environment; (ii) a quantitative description of the laws of optical flow allowing visualisation of the changing visual scene associated with progression along any pathway in the 3D environment; (iii) object recognition and ability to correct for changes in perceived size and shape as a function of position and orientation of objects in the environment relative to the observer; (iv) ability to see the world from another person’s point of view and ability to acquire new motor skills through imitation and mental imagery; and (v) ability to visualise a familiar environment as if seen from a place not previously experienced enabling, for example, the drawing of a furniture layout as if seen from a point above the room.

As set out previously in [2], the theory addresses a similar range of issues in action science. However, most importantly, that paper provides a new way of linking perception and action, taking into account the redundancies of multi-joint movement and the nonlinear warping of both visual space and proprioceptive space. The formulation yields neurally feasible computational processes for selecting minimum-effort movement synergies to achieve specified visual goals during natural behaviours. This is a contribution that has not to our knowledge been achieved elsewhere. Central to that proposal is the visual–somatosensory–hippocampal memory that we draw on in this paper to analyse and explain the experimentally observed dissociations between perception and action in the investigation of afterimages and illusions. This provides cohesion between diverse reports as well as giving support to the theory.

In conclusion, we believe that Riemannian geometry provides the most suitable mathematical framework for the analysis of the many nonlinear dynamical processes involved in both visual perception and movement control and in their integration. However, in keeping with Popper [28], we are aware that a long list of experimental findings consistent with the predictions of a theory does not prove the theory. It is always possible that the very next experiment will show the theory to be wrong. Refutation is a necessary key to enhancing knowledge. Nevertheless and meanwhile, we contend that the many findings addressed in this paper and accounted for in a single framework are illustrative of the predictive power of the Riemannian geometry theory of visually-guided movement. 

## Data Availability

Not applicable.
